# Correlation analysis between the severity of respiratory syncytial virus pneumonia and the expression levels of inflammatory cytokines in bronchoalveolar lavage fluid among infants and young children

**DOI:** 10.3389/fped.2025.1482029

**Published:** 2025-02-19

**Authors:** Lin Tan, Zhaohui He, Yongqi Liang, Kuncheng Wang, Xiaoqian Chen

**Affiliations:** ^1^ Department of Pediatrics, The First People’s Hospital of Foshan, Foshan, Guangdong, China; ^2^ Clinical Laboratory, Nanhai District People’s Hospital of Foshan, Foshan, Guangdong, China

**Keywords:** infants and young children, respiratory syncytial virus pneumonia, bronchoalveolar lavage fluid, inflammatory cytokines, correlation analysis

## Abstract

**Purpose:**

RSV is the primary cause of lower respiratory tract infections in infants and young children. Current study aims to investigate the correlation between the severity of respiratory syncytial virus pneumonia (RSVP) in infants and young children and the number of RSV infection sequences as well as the levels of cytokines in bronchoalveolar lavage fluid (BALF).

**Methods:**

Metagenomics next-generation sequencing (mNGS) and enzyme-linked immunosorbent assay (ELISA) were used to detect the number of RSV infection sequences and the levels of related inflammatory cytokines in BALF samples. Comparisons between groups and Logistic regression analysis were performed to examine the differences in RSV infection sequences and inflammatory cytokine levels between the sRSVP and nsRSVP groups. Spearman's correlation coefficient was used to analyze the correlations among PCIS, RSV infection sequences, and inflammatory cytokines. Finally, ROC curve analysis was conducted to assess the diagnostic performance of inflammatory cytokines as biomarkers in determining the severity of RSVP.

**Results:**

A total of 49 infants and young children diagnosed with RSV infection were enrolled and divided into severe RSVP (sRSVP) and non-severe RSVP (nsRSVP) groups based on the pediatric critical illness score (PCIS) scale. The levels of Interleukin (IL)-6, IL-8, IL-10, tumor necrosis factor α (TNF-α), IL-17A, and monocyte chemotactic protein 1 (MCP-1) as well as the number of RSV sequences in BALF were significantly higher in the sRSVP group than in the nsRSVP group. Additionally, elevated levels of IL-6, IL-10, TNF-α, IL-17A, and the number of RSV sequences were identified as risk factors for the severity of RSVP. Spearman's correlation analysis revealed significant negative correlations between the levels of IL-6, IL-10, TNF-α, IL-17A, and MCP-1 in BALF with PCIS, and significant positive correlations with the number of RSV sequences. Furthermore, a significant negative correlation was observed between RSV sequence count and PCIS. ROC curve analysis showed that the levels of IL-6, IL-10, TNF-α, IL-17A, and MCP-1, as well as their combined diagnostic approach, exhibited high diagnostic performance in determining the severity of RSVP.

**Conclusion:**

The levels of inflammatory cytokines and RSV sequences in BALF are significantly correlated with the severity of RSVP in infants and young children. The levels of IL-6, IL-10, TNF-α, IL-17A, and MCP-1 can serve as potential biomarkers for diagnosing the severity of RSVP.

## 1 Introduction

Respiratory syncytial virus (RSV) is a single-stranded, negative-sense RNA virus and the predominant viral pathogen responsible for acute lower respiratory tract infections in infants and young children (1). RSV is the leading cause of such infections in this demographic, affecting over 70% of infants under one year of age and nearly all children by the age of two worldwide (2). The virus can induce a spectrum of diseases, including bronchiolitis and pneumonia, and may lead to mortality, affecting both the upper and lower respiratory tracts (3). Globally, RSV-related infections result in approximately 20.8 million cases, 1.8 million hospitalizations, 40,000 deaths, 1.2 million disability-adjusted life years, and incur healthcare costs amounting to USD 611 million across 72 countries (4). Severe early RSV infections may have adverse effects on lung development, leading to chronic respiratory diseases such as recurrent wheezing after infection (5). Only a parent vaccine and a long-acting monoclonal antibody have recently been approved for prevention or improvement of RSV disease and severity in some countries (6). However, effective RSV vaccines and antiviral treatments are not yet widely implemented (7). To effectively control the spread of RSV, the World Health Organization (WHO) established a global RSV surveillance network in 2017 and included RSV vaccines in the list of priority infectious disease vaccines for global development (8).

Despite the high incidence of RSV infections, its pathogenesis remains unclear. One of the main reasons hindering the development of RSV antiviral drugs is the lack of evidence showing that higher RSV loads correlate with more severe disease manifestations (9). However, there is inconsistency in some research results. One study found that RSV viral loads in outpatient infants with mild RSV infections were higher than those in hospitalized infants with severe RSV infections (10). Additionally, Mazur et al. found no correlation between RSV viral loads and disease severity in RSV-positive children (11). Conversely, a study by Erika et al., which included 201 RSV-infected children, demonstrated that higher viral loads were associated with longer symptom duration (9). To our knowledge, this is the largest analysis to date assessing the relationship between RSV loads and clinical disease in outpatient children (12). Similarly, Houben et al. found a positive correlation between RSV loads and disease severity scores in 30 infants with community-acquired RSV infections (12). If viral load is a major factor influencing clinical RSV disease, then reducing viral load through antiviral interventions could potentially improve outcomes. However, the relationship between RSV infection abundance and the severity of RSV pneumonia (RSVP) remains ambiguous. In recent years, metagenomics next-generation sequencing (mNGS) has gradually demonstrated its advantages and potential in the etiological diagnosis of respiratory tract infections (13, 14). Therefore, we aim to explore the correlation between RSV infection abundance and RSVP severity by measuring RSV infection sequence numbers in bronchoalveolar lavage fluid (BALF) using mNGS.

Understanding the clinical characteristics of RSVP and assessing infection severity are crucial for treatment and prognosis. RSV, a common respiratory pathogen, interacts with monocyte CD14 and Toll-like receptors, triggering nuclear factor-κB (NF-κB) activation and inflammatory cytokine release, causing systemic inflammation (15, 16). However, research in this area is still limited. The relationship between inflammatory markers and disease severity in RSV-infected children has not yet been clearly defined. Therefore, we further analyzed the correlation between different inflammatory factor levels in BALF and disease severity, while exploring the diagnostic value of inflammatory factors in the severity of RSV infection. This offers new insights for early prediction of severe pneumonia in infants and young children.

## 2 Materials and methods

### 2.1 Study subjects

Children hospitalized with RSVP in the Children's intensive care unit of Foshan First People's Hospital from May 2023 to May 2024 were selected as the study objects. Inclusion criteria were: ① confirmed RSV infection; ② age ranging from 29 days to 3 years; ③ all children underwent bronchoscopy and obtained bronchoalveolar lavage fluid (BALF) samples; ④ RSV was detected in BALF samples through metagenomic next-generation sequencing (mNGS); ⑤ the children's families were informed and signed the consent form. Exclusion criteria were: ① co-infection with bacteria or fungi; ② congenital heart disease; ③ chronic lung disease; ④ immune deficiency; ⑤ children who received systemic steroid treatment within 2 weeks before onset. Withdrawal criteria were: ① children requesting transfer during hospitalization; ② children requesting withdrawal during the study.

Based on the inclusion and exclusion criteria, a total of 49 patients with RSVP were included in this study. The severity of RSVP was evaluated using the pediatric critical illness score (PCIS) scale (16). Children with PCIS ≤ 80 were included in the severe RSVP group (sRSVP, 23 cases), and those with PCIS > 80 were included in the non-severe RSVP group (nsRSVP, 26 cases). The PCIS scoring criteria are provided in the Supplementary Materials (Supplementary Table S1).

### 2.2 Collection of clinical data

Fiberoptic bronchoscopy and BALF sample collection were performed within 24 h of admission for all children. Normal saline was injected into the diseased lung segment or sublung segment, the total amount was 3–5 ml per kg of body weight, and the recovery rate was not less than 30%. Finally, 5 ml of BALF was collected, stored at 4°C, and sent for testing within 2 h. Of this, 3 ml of BALF was used for mNGS detection, and 2 ml was used for cytokine detection.

#### 2.2.1 mNGS detection

Total nucleic acid was extracted from BALF samples using the QIAamp DNA Mini Kit (Qia-gen, Germany). The NEBNext Ultra II DNA Library Prep Kit for Illumina (NEB, USA) was used to construct the libraries. Then, high-throughput sequencing was performed using the Illumina NovaSeq 6,000 platform with paired-end 150 bp reads. After sequencing, the data underwent quality control, removal of human sequences, low-quality sequences, and duplicate sequences. Subsequently, the data were compared and analyzed with the NCBI database. The number of RSV infection sequences was determined based on the alignment results. The detection threshold was set at a minimum of 5 reads per million reads matching the target sequence.

#### 2.2.2 Enzyme-linked immunosorbent assay (ELISA) detection

The levels of Interleukin (IL)-2, IL-4, IL-6, IL-8, IL-10, IL-17A, tumor necrosis factor α (TNF-α), Interferon γ (IFN-γ), monocyte chemotactic protein 1 (MCP-1), and C-reactive protein (CRP) in BALF from children with RSVP were detected using enzyme-linked immunosorbent assay (ELISA). Briefly, BALF samples were centrifuged at 1,500 rpm/min for 15 min. Inflammatory factor testing of centrifuged supernatants was performed following the manufacturer's instructions associated with the ELISA kit (Thermo Fisher Scientific, Massachusetts, USA). Absorbance readings were obtained at a wavelength of 570 nm by CLARIOstar microplate reader (BMG LABTECH, Germany).

### 2.3 Statistical analysis

Data analysis was performed using SPSS 27.0 software. The Kolmogorov–Smirnov test was first used to determine whether the measurement data conformed to a normal distribution. Normally distributed measurement data were expressed as Mean ± SD. Non-normally distributed measurement data were expressed as median (interquartile range). Independent sample T-test was used for the comparison of normally distributed measurement data between groups. The Kolmogorov–Smirnov test was used for the comparison of non-normally distributed measurement data between groups. Binary Logistic regression was used for influencing factor analysis. Spearman's correlation analysis was used to assess the correlation between two linear indicators. The diagnostic efficacy of single and combined indicators was analyzed using the receiver operating characteristic (ROC) curve. The confidence interval was set at 95%, and *P* < 0.05 was considered statistically significant.

## 3 Results

### 3.1 Analysis of inflammatory factor levels in BALF of patients with RSVP

Patients with RSVP were divided into the severe RSVP group (sRSVP, PCIS ≤ 80, 23 cases) and the non-severe RSVP group (nsRSVP, PCIS > 80, 26 cases) based on the Pediatric Clinical Illness Severity (PCIS) score. Of these, patients in the sRSVP group were 17 ± 11 months old, 14 males and 9 females; patients in the nsRSVP group were 20 ± 11 months old, 15 males and 11 females. Age and sex were not statistically different between the sRSVP and nsRSVP groups. As shown in Table 1, a comparison of the inflammatory factor levels and RSV sequence numbers in BALF between the sRSVP and nsRSVP groups revealed that the levels of IL-6, IL-8, IL-10, TNF-α, IL-17A, and MCP-1, as well as the RSV sequence numbers, were significantly elevated in the sRSVP group compared to the nsRSVP group (All *P* < 0.05). In addition, there were no statistically significant differences in the levels of IL-2, IL-4, IFN-γ, and CRP in BALF between the sRSVP and nsRSVP groups (All *P* > 0.05). Furthermore, the RSV sequence number detected in BALF samples from the sRSVP group was significantly higher than that in the nsRSVP group (*P* < 0.001).

**Table 1:** Comparison of inflammatory factor levels in BALF of patients with RSVP with different severity levels.

Cytokines	sRSVP (*n* = 23)	nsRSVP (*n* = 26)	t/Z	*P*
IL-2	1.10 (0.60, 1.40)	1.55 (0.55, 2.00)	1.122	0.161
IL-4	1.27 ± 0.70	1.45 ± 0.70	−0.905	0.370
IL-6	709.50 (242.20, 2,265.70)	38.25 (10.65, 97.03)	2.652	<0.001
IL-8	97.47 ± 32.92	80.59 ± 24.85	2.041	0.047
IL-10	13.40 (3.80, 52.80)	1.85 (1.20, 5.68)	2.027	<0.001
TNF-α	49.60 (4.40, 152.00)	2.25 (1.78, 53.25)	1.595	0.012
IFN-γ	10.60 (2.60, 131.80)	6.80 (1.78, 40.38)	0.835	0.488
IL-17A	29.10 (8.90, 108.10)	5.45 (4.00, 8.35)	2.483	<0.001
MCP-1	29.30 (24.20, 42.60)	19.05 (13.48, 22.48)	2.348	<0.001
CRP	16.37 ± 8.76	12.07 ± 7.74	2.164	0.075
Number of sequences	280 (88, 651)	56 (24, 74)	2.448	<0.001

IL-2, IL-6, IL-10, TNF-α, IFN-γ, IL-17A, MCP-1 and Number of sequences are shown as Median with IQRs; IL-4, IL-8 and CRP are shown as Mean ± SD.

**Figure 1:**
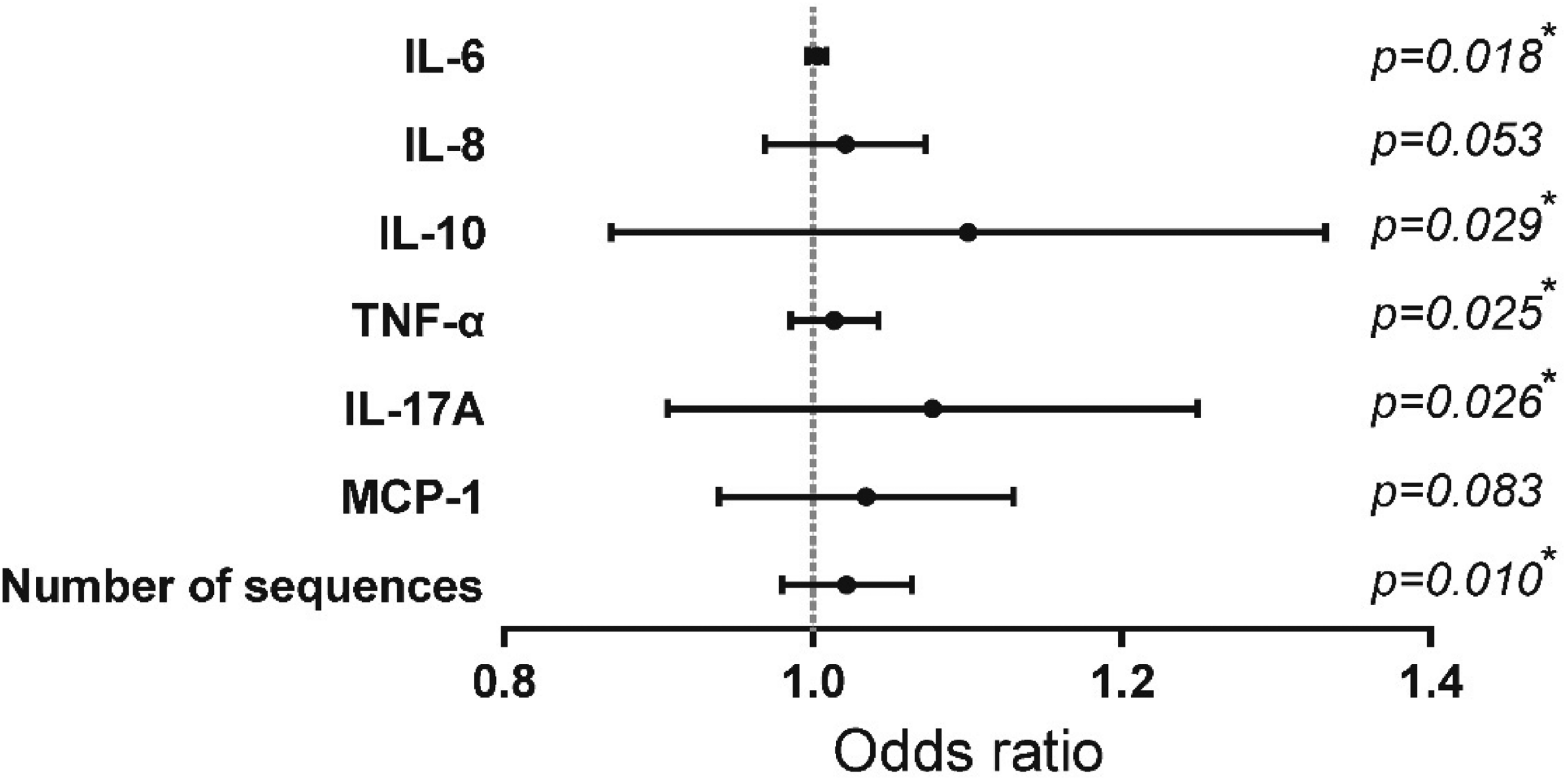
Risk factor analysis for the severity of RSVP.

Candidate variables with significant differences in univariate analysis were included in a univariate Logistic regression analysis to further explore the risk factors for the severity of RSVP, as presented in Table 2 and Figure 1. Among the inflammatory factors, IL-6 (OR = 1.003), IL-10 (OR = 1.098), TNF-α (OR = 1.013), and IL-17A (OR = 1.076) were identified as risk factors for the development of severe RSVP. An increase in the levels of IL-6, IL-10, TNF-α, or IL-17A by 1 pg/ml was associated with an increased risk of severe RSVP by 0.3%, 9.8%, 1.3%, or 7.6%, respectively. Additionally, RSV sequence number (OR = 1.022) was also a risk factor for severe RSVP, with an increased risk of 2.2% for each additional RSV sequence number. However, further analysis using multivariate Logistic regression (after adjusting for confounders) revealed that the significant effects of the above-mentioned candidate variables on the risk of severe RSVP disappeared (Data not shown), suggesting that these factors were not independent risk factors for severe RSVP. The occurrence of severe RSVP is a result of the interaction between changes in different inflammatory factor levels and RSV infection abundance.

**Table 2:** Logistic regression analysis of factors influencing the severity of RSVP.

Variable	B	SE	WaldX^2^	*P*	OR	95%CI
IL-6	0.003	0.001	5.554	0.018	1.003	1.000–1.005
IL-8	0.021	0.011	3.732	0.053	1.021	1.000–1.042
IL-10	0.094	0.043	4.741	0.029	1.098	1.009–1.195
TNF-α	0.013	0.006	5.022	0.025	1.013	1.002–1.025
IL-17A	0.073	0.033	4.949	0.026	1.076	1.009–1.147
MCP-1	0.033	0.019	3.015	0.083	1.034	0.996–1.073
Number of sequences	0.022	0.009	6.635	0.010	1.022	1.005–1.039

### 3.2 Correlation analysis of RSVP severity, RSV sequence numbers, and BALF inflammatory factor levels

**Figure 2:**
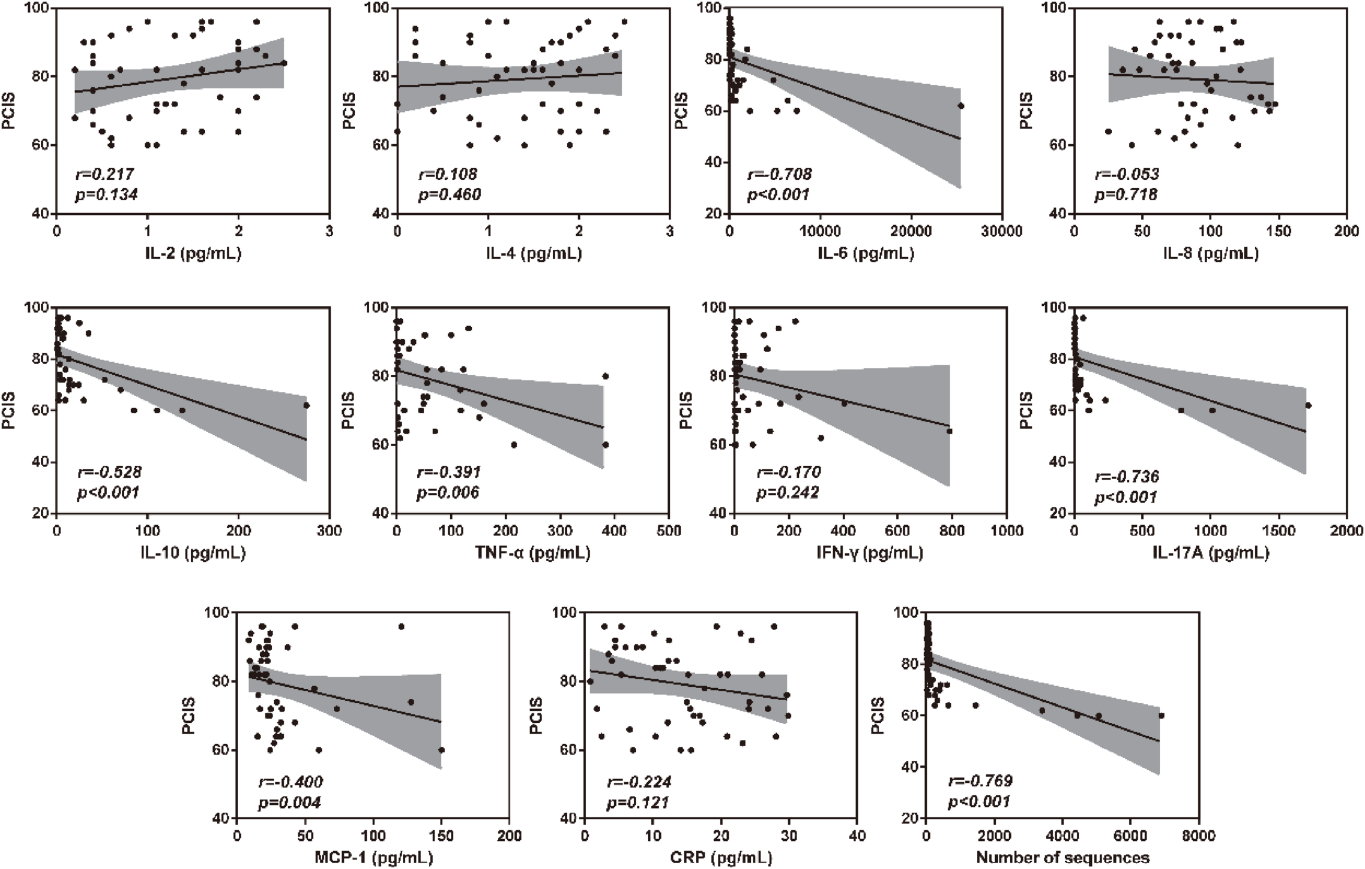
Clinical correlation analysis between BALF inflammatory factor levels and PCIS.

**Figure 3:**
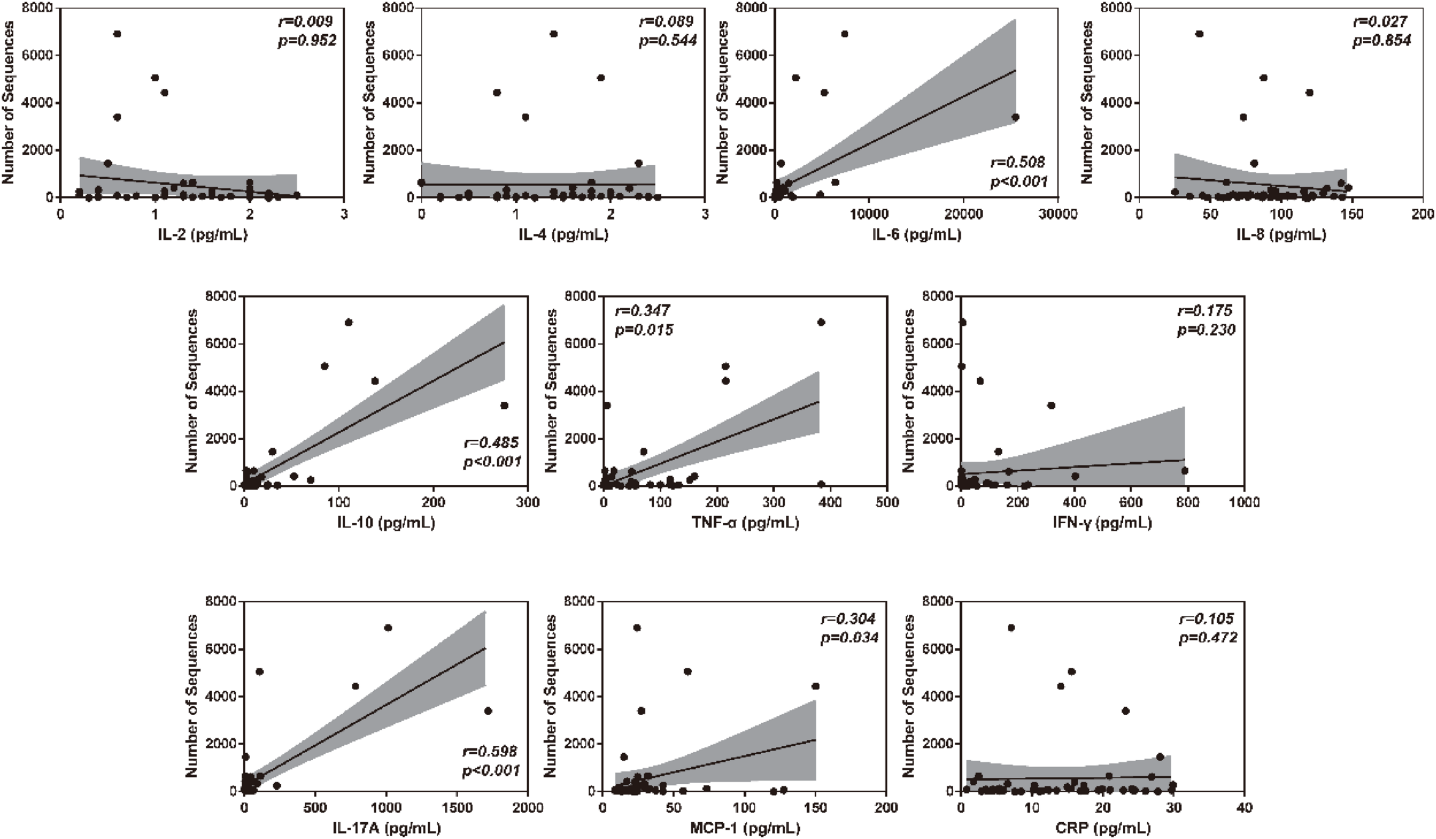
Clinical correlation analysis between BALF inflammatory factor levels and RSV sequence numbers.

To verify the association between inflammatory factor levels and the severity of RSVP, the correlation between BALF inflammatory factor levels and PCIS was evaluated using Spearman's correlation analysis. As shown in Figure 2, the levels of IL-6, IL-10, TNF-α, IL-17A, and MCP-1 in BALF were significantly negatively correlated with PCIS (All r < 0, *P* < 0.05). This indicates that an increase in the levels of IL-6, IL-10, TNF-α, IL-17A, and MCP-1 in BALF may suggest more severe lung function impairment. Additionally, the levels of IL-2, IL-4, IL-8, IFN-γ, and CRP showed no significant correlation with PCIS (*P* > 0.05). As expected, there was also a significant negative correlation between RSV sequence numbers and PCIS (r < 0, *P* < 0.001) (Figure 2). Therefore, we further analyzed the correlation between RSV sequence numbers and different inflammatory factor levels in BALF. As illustrated in Figure 3, the levels of IL-6, IL-10, TNF-α, IL-17A, and MCP-1 in BALF were significantly positively correlated with RSV sequence numbers (All r > 0, *P* < 0.05). Meanwhile, the levels of IL-2, IL-4, IL-8, IFN-γ, and CRP showed no significant correlation with RSV sequence numbers (*P* > 0.05).

### 3.3 Application value of BALF inflammatory factor levels in the diagnosis of RSVP severity

**Figure 4:**
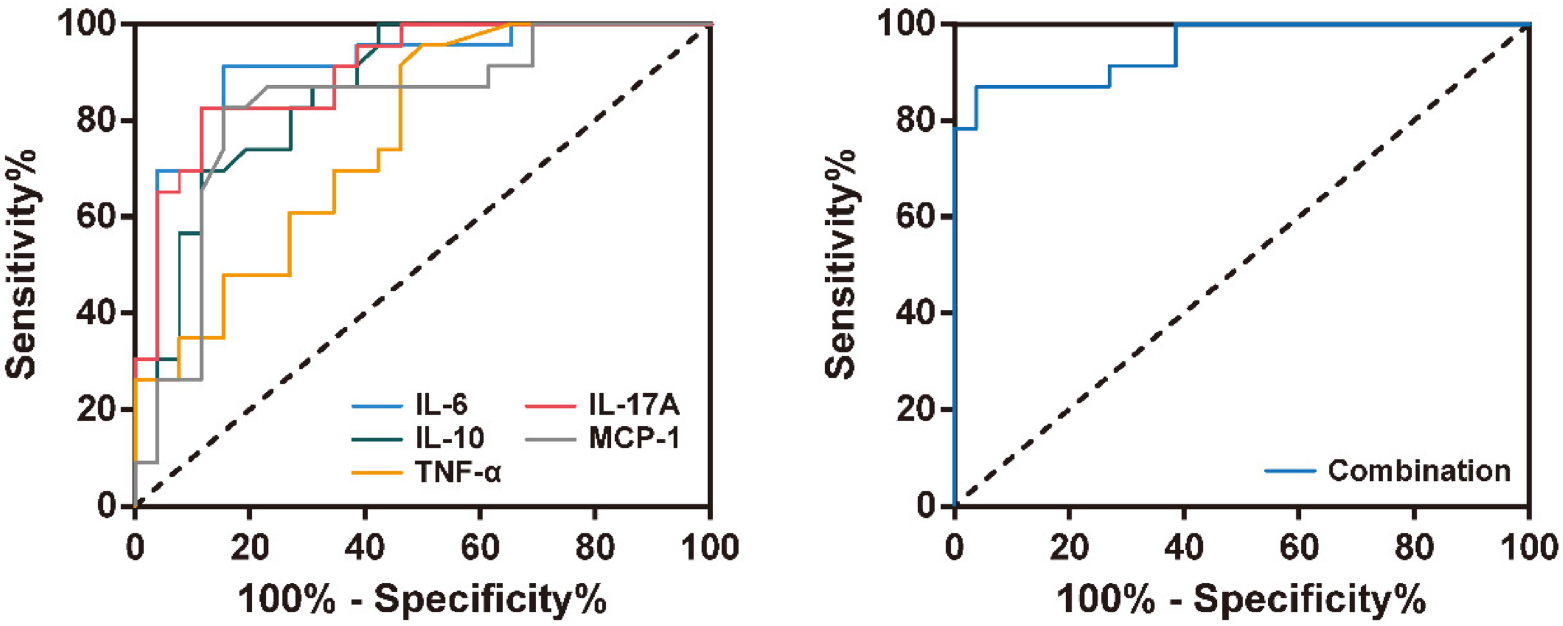
ROC curve analysis of BALF inflammatory factor levels in the diagnosis of RSVP severity.

To evaluate the diagnostic value of inflammatory factors in BALF for RSVP severity, we selected inflammatory factors significantly associated with RSVP severity for ROC curve analysis. As shown in Figure 4 and Table 3, the levels of IL-6, IL-10, TNF-α, IL-17A, and MCP-1 all exhibited good predictive value for RSVP severity (All *P* < 0.001). Specifically, RSVP severity was high when IL-6 ≥ 188.30 pg/ml, IL-10 ≥ 8.60 pg/ml, TNF-α ≥ 2.25 pg/ml, IL-17A ≥ 8.75 pg/ml, or MCP-1 ≥ 23.80 pg/ml. Among these, IL-6 (AUC = 0.910) was the best cytokine for distinguishing severe RSVP (sRSVP) from non-severe RSVP (nsRSVP) in infants and young children. Additionally, the diagnostic sensitivity of IL-6 and TNF-α for sRSVP was over 90%, indicating a low missed diagnosis rate. However, the diagnostic specificity of TNF-α was only 50.0%, suggesting a high misdiagnosis rate. Further analysis of the combined diagnostic ROC curve of these five cytokines revealed that the combined diagnosis had a slightly higher diagnostic efficacy (AUC = 0.952) than any single diagnostic method, with a diagnostic sensitivity and specificity of 87.0% and 96.2%, respectively.

**Table 3:** Diagnostic efficacy of BALF inflammatory factor levels in RSVP severity.

Cytokines	AUC (95% CI)	Cut-off value	Sensitivity	Specificity	*P* value
IL-6	0.910 (0.825, 0.995)	188.30 pg/ml	91.3%	84.6%	<0.001
IL-10	0.866 (0.767, 0.966)	8.60 pg/ml	69.6%	88.5%	<0.001
TNF-α	0.763 (0.631, 0.894)	2.25 pg/ml	95.7%	50.0%	<0.001
IL-17A	0.901 (0.817, 0.985)	8.75 pg/ml	82.6%	88.5%	<0.001
MCP-1	0.827 (0.703, 0.950)	23.80 pg/ml	82.6%	84.6%	<0.001
Combination	0.952 (0.896, 1.000)	/	87.0%	96.2%	<0.001

## 4 Discussion

The Pediatric Critical Illness Score (PCIS) uses 10 quantitative indicators from lab tests or physical exams to assess the severity and prognosis of critically ill children (17). Few studies explore the link between RSVP and PCIS, and early indicators for RSVP severity are lacking. This study included 49 patients with RSVP and divided them into 23 cases of sRSVP (PCIS ≤ 80) and 26 cases of nsRSVP (PCIS > 80) based on PCIS. A retrospective analysis was conducted on the levels of inflammatory factors and RSV infection sequences in BALF between the two groups, systematically evaluating the clinical characteristics of RSVP children and their correlation with PICS scores. The results showed that the levels of IL-6, IL-8, IL-10, TNF-α, IL-17A, and MCP-1 in BALF of patients with sRSVP were significantly higher than those in patients with nsRSVP. Further univariate Logistic regression analysis revealed that IL-6, IL-10, TNF-α, and IL-17A were risk factors for promoting the occurrence of sRSVP.

After RSV infection, the body is in a dynamic state of immune response and inflammatory stress (18). The expression of proinflammatory cytokines is a related immunopathological feature after RSV infection (19). The exacerbated inflammation of neutrophils and lymphocytes is thought to trigger an unbalanced pathogenic helper T (Th)2 response (19). During RSV infection, the Th1 response is a proinflammatory response that reduces the production of chemokines CCL11, CCL17, and CCL22 by secreting Th1-type cytokines such as TNF-α and IFN-γ, thereby inhibiting the accumulation of eosinophils in the lungs (20). However, this study did not observe significant differences in IFN-γ levels between patients with sRSVP and nsRSVP. Th2 cells exert their effects through the production of Th2-type cytokines (21). The Th-2 response is characterized by the production of inflammatory factors such as IL-4, IL-6, and IL-10, which participate in the generation of antibodies (especially IgE) and eosinophil responses (5). The rapid production of IL-6 contributes to host defense during infection (22, 23). In addition, IL-8 can promote neutrophils to produce inflammatory mediators, stimulate eosinophils, and exacerbate airway inflammation (24). IL-17A is an effector cytokine of the innate and adaptive immune systems involved in host defense and maintenance of tissue integrity (25). A study by Long et al. found a strong positive correlation between IL-17A levels and RSV infection severity (25). Furthermore, multiple studies have shown that the concentration of IL-17A in BALF of RSV-infected infants is higher than that in the control group (26, 27), especially in critically ill children (28). Additionally, MCP-1 plays an important role in lung inflammation by enhancing Th2 polarization and monocyte attraction to the lungs (29). Consistent with these findings, we observed a significant negative correlation between the levels of IL-6, IL-8, IL-10, TNF-α, MCP-1, and IL-17A in BALF and PCIS. Moreover, studies have shown that the percentage of mononuclear macrophages in BALF also increases during RSV infection (30). Alveolar macrophages detect RSV viral antigens and release cytokines and chemokines early in RSV infection (31). Alveolar macrophages exposed to RSV *in vitro* produce IL-6, TNF-α, and IL-10 within 20 h after exposure, and these levels increase with increasing viral doses. In addition, Song et al. confirmed elevated IL-6, IL-10, and MCP-1 levels in BALF post-RSV infection (32). Similarly, this study showed that these cytokine levels were significantly positively correlated with RSV infection sequences.

Most previous studies have examined the relationship between RSV load and disease severity by measuring disease severity using various outcomes. Up to now, although several studies have found a positive correlation between RSV load and disease severity, including the largest sample size studies (9, 33, 34), many studies have not reached the same conclusion (35–37). We believe that there are many potential reasons for the inconsistent results, such as small sample size or low sensitivity in determining the severity of the disease. Therefore, this study is the first to incorporate the Pediatric Critical Illness Score (PCIS) as an indicator to measure the severity of RSV infection. PCIS assesses critically ill pediatric cases by measuring 10 quantitative indicators, all of which are quantitative data or objective indicators obtained from laboratory tests or physical examinations, and can accurately reflect the severity of pediatric diseases (38). In this study, the number of RSV sequences detected in BALF of patients in the sRSVP group was significantly higher than that in patients with nsRSVP. Univariate Logistic regression analysis showed that RSV sequence number was also a risk factor for promoting the occurrence of critical RSVP. Moreover, there was a significant negative correlation between RSV sequence number and PCIS, indicating that the higher the abundance of RSV, the higher the severity of RSVP. Similarly, the levels of IL-6, IL-10, TNF-α, IL-17A, and MCP-1 in BALF of patients with RSVP were positively correlated with RSV sequence numbers. In summary, this association suggests a mechanism that reducing RSV infection abundance through therapeutic intervention can further reduce the severity of RSVP. Theoretically, the use of effective RSV antiviral drugs may reduce the severity of RSVP.

We have demonstrated that PCIS can be used as a diagnostic indicator for measuring the severity of RSVP. Additionally, we found significant correlations between the levels of IL-6, IL-10, TNF-α, IL-17A, and MCP-1 in BALF of patients with RSVP and the severity of RSVP, potentially serving as auxiliary diagnostic indicators for RSVP severity. The ROC analysis results showed that the levels of IL-6, IL-10, TNF-α, IL-17A, and MCP-1 all had good predictive value for the severity of RSVP, effectively distinguishing sRSVP from nsRSVP. Furthermore, the combined diagnosis of these five inflammatory factors further increased the diagnostic efficiency for RSVP severity, with a diagnostic sensitivity and specificity of up to 87.0% and 96.2%. These results suggest that IL-6, IL-10, TNF-α, IL-17A, and MCP-1 in BALF can serve as effective biomarkers for the severity of RSVP in children.

This study has the following limitations. Firstly, the sample size is relatively small, which may introduce potential bias. Secondly, all the study samples were hospitalized children or patients in the Pediatric Intensive Care Unit (PICU). Since the conditions of hospitalized and patients in PICU are more severe than those of general outpatient children, the results may not be applicable to the general outpatient setting. Additionally, due to the incomplete plasma samples of the included patients, this study did not compare the differences in cytokine levels and RSV infection abundance between plasma and BALF to ensure consistency in sample research results. Finally, due to the difficulty in obtaining BALF samples from healthy infants and young children, this study lacked a healthy control group. However, we still obtained reliable conclusions that can assist clinicians in analyzing the characteristic differences between severe RSV pneumonia (sRSVP) and non-severe RSV pneumonia (nsRSVP). Finally, the list of inflammatory markers in this study is limited, and further investigation of the association between other inflammatory factors and the severity of illness in children with RSV infection is still needed subsequently. This study is the first to reveal the relationship between RSVP severity levels, RSV sequence numbers, and BALF inflammatory factor. In addition, this study further analyzed the diagnostic efficacy of BALF inflammatory factor levels in RSVP severity. These have practical reference significance for timely and accurate identification and diagnosis of clinical infants and young children with RSVP and subsequent prevention and treatment.

## 5 Conclusion

In summary, we have demonstrated the correlation between the levels of IL-6, IL-10, TNF-α, IL-17A, and MCP-1 in BALF and the severity of RSV pneumonia. Furthermore, this study provides support for the concept that RSV infection abundance drives the severity of RSV pneumonia in infants and young children. The development of RSV antiviral drugs to reduce RSV infection abundance may help mitigate the severity of RSV pneumonia in patients. Finally, the levels of IL-6, IL-10, TNF-α, IL-17A, and MCP-1 can serve as biomarkers for the severity of RSV pneumonia, which is conducive to early diagnosis and subsequent personalized management and treatment for RSV-infected children.

## Data Availability

The original contributions presented in the study are included in the article/Supplementary Material, further inquiries can be directed to the corresponding author.
