# Supplementary material for: Correlation analysis between the severity of respiratory syncytial virus pneumonia and the expression levels of inflammatory cytokines in bronchoalveolar lavage fluid among infants and young children
Source: Front Pediatr. 2025 Feb 19;13:1482029. doi: 10.3389/fped.2025.1482029 (PMC11880017; doi:10.3389/fped.2025.1482029)
Supplement: Supplementary file 1 [file Table1.docx]

**Table S1 Pediatric Critical Illness Score, PCIS.**

| **Inspection item** | **Measured Value and Performance** | | **Score** |
| --- | --- | --- | --- |
|  | Age < 1 year | Age ≥ 1 year |  |
| Heart rate (times/min) | < 80 or > 180 | < 60 or > 160 | 4 |
|  | 80~100 or 160~180 | 60~80 or 140~160 | 6 |
|  | Other | Other | 10 |
| Systolic blood pressure  mmHg (kpa) | < 55 (7.3) or > 130 (17.3) | < 65 (8.7) or > 150 (20.0) | 4 |
|  | 55~65 (7.3~8.7) or 100~130 (13.3~17.3) | 65~75 (8.7~10.0) or 130~150 (17.3~20.0) | 6 |
|  | Other | Other | 10 |
| Breathing (cycles per minute) | < 20 or > 70 Obvious irregular rhythm | < 15 or > 60 Obvious irregular rhythm | 4 |
|  | 20~25 or 40~70 | 15~20 or 35~60 | 6 |
|  | Other | Other | 10 |
| PaO_2_ mmHg (kpa) | < 6.7 (50) | | 4 |
|  | 6.7~9.3 (50~70) | | 6 |
|  | Other | | 10 |
| PH | < 7.25 or > 7.55 | | 4 |
|  | 7.25~7.30 or 7.50~7.55 | | 6 |
|  | Other | | 10 |
| Na^+^ mmol/L | < 120 or > 160 | | 4 |
|  | 120~130 or 150~160 | | 6 |
|  | Other | | 10 |
| K^+^ mmol/L | < 3.0 or > 6.5 | | 4 |
|  | 3.0~3.5 or 5.5~6.5 | | 6 |
|  | Other | | 10 |
| Cr μmol/L (mg/dL) | >159 (1.8) | | 4 |
|  | 106~159 (1.2~1.8) | | 6 |
|  | Other | | 10 |
| BUN mmol/L (mg/dL) | >14.3 (40) | | 4 |
|  | 7.1~14.3 (20~40) | | 6 |
|  | Other | | 10 |
| Hb g/L (g/dL) | <60 (6) | | 4 |
|  | 60-90 (6~9) | | 6 |
|  | Other | | 10 |
| Gastrointestinal system | Stress ulcer and intestinal paralysis | | 4 |
|  | Bleeding from stress ulcer | | 6 |
|  | Other | | 10 |

PCIS > 80: non-critical；PCIS ≤ 80: critical
